# Metamorphic sulfur release as a driver of sustained cooling and mass extinction

**DOI:** 10.1126/sciadv.aee2277

**Published:** 2026-07-08

**Authors:** Emily M. Stewart, Michael S. Diamond, Lindsi J. Allman

**Affiliations:** Department of Earth, Ocean & Atmospheric Science, Florida State University, Tallahassee, FL, USA.

## Abstract

The emplacement of large igneous provinces may drive catastrophic volatile release, both directly through volcanic degassing and indirectly through heating of carbon- and sulfur-bearing host sediments. It is broadly assumed that sulfur injection must reach the stratosphere to drive long-term cooling; thus, these indirect metamorphic sulfur emissions have been almost entirely ignored. Here, we demonstrate that plausible carbon and sulfur emissions from contact metamorphism may be sustained long enough to cause centennial-scale sulfate aerosol–driven cooling spikes of several kelvins superimposed on millennial-scale warming from the carbon dioxide greenhouse effect. We use modeling of sediment metamorphism along with simple carbon cycle and planetary energy balance models of the climate response to explore this relationship. Our results suggest that a metamorphic sulfur source should be considered as a driver of sustained global cooling during large igneous province emplacement, with potential implications for Phanerozoic biotic crises such as the End-Triassic Mass Extinction.

## Teaser

Ancient mass extinction events may result from metamorphic sulfur release, driving catastrophic global winters lasting centuries.

## INTRODUCTION

**Fig. 1.**
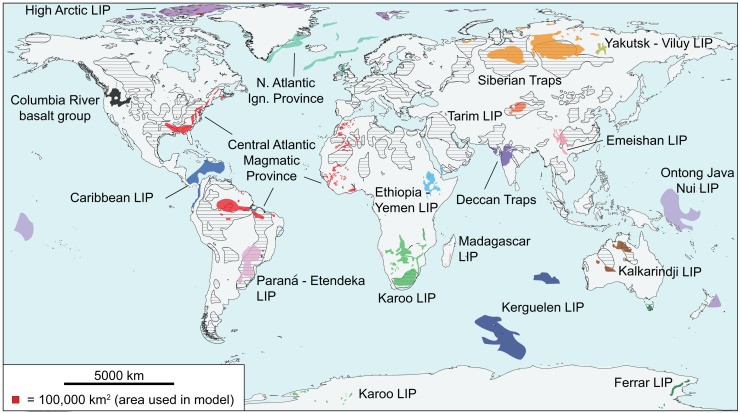
LIPs and shale basins. This equal-area global map shows the locations of major LIPs indicated in color [after (*58*)]. Shale basins are indicated with horizontal stripes and locations are from (*59*).

Large igneous provinces (LIPs) have been accused of driving many of Earth’s most catastrophic mass extinction events (*1*–*3*). These massive intrusive and extrusive complexes cover an area of more than 100,000 km^2^ and release a correspondingly large mass of volatiles into the ocean-atmosphere system (Fig. 1) (*4*). These volatiles and their respective climatic effects are suggested as mechanisms for driving extinction: Carbon in the form of carbon dioxide (CO_2_) or methane (CH_4_) acts as a greenhouse gas, insulating and warming the planet, while sulfur in the form of aerosols [e.g., sulfate (SO_4_) produced from the atmospheric oxidation of sulfur dioxide (SO_2_)] reflects sunlight directly and indirectly via changing cloud properties, producing regional or global cooling. Whiplash between warming and cooling events may be more likely to cause extinction than monotonic warming or cooling on its own (*5*–*8*).

LIPs may release volatiles directly through volcanic eruptions or degassing of magma chambers. Recently, many researchers have also considered indirect volatile release: When hot basaltic magma intrudes into labile volatile-rich sediments, the resulting contact metamorphism has the potential to degas massive amounts of material (*9*–*13*). The geologic community has historically focused on metamorphic release of carbon-bearing greenhouse gases with only a handful of papers considering metamorphic sulfur release (*13*–*15*).

This neglect may stem in part from a common oversimplification: It is often suggested (*13*, *16*–*18*) that sulfur release into the troposphere will have a negligible effect on global climate on multiyear timescales because the residence time of sulfur species in the troposphere is vanishingly small in a geologic context, on the order of days to weeks. The geologic community, which historically focused on functionally instantaneous volcanic degassing, thus generally assumes that low-altitude emissions into the troposphere do not act on long timescales. This may be a very appropriate assumption for a time-limited volcanic injection. However, the duration of the emissions themselves will have a profound effect: If emissions are sustained over many years, the corresponding climate response will be similarly prolonged. For example, Schmidt *et al.* (*19*) used a global aerosol model to simulate upper tropospheric sulfur emissions from decadal-scale flood basalt eruptions, concluding that sustained, centennial-scale sulfur releases would be required for tropospheric aerosol cooling to be a plausible factor in LIP-associated mass extinction. On the modern Earth, low-altitude sulfur emissions, e.g., from modern cargo shipping (*20*–*22*) and effusive volcanism (*23*, *24*), produce a measurable brightening effect on marine clouds and, thus, negative climate forcing over multiple years. Overall, cooling from anthropogenic aerosol emissions in the lower troposphere is currently assessed to offset approximately one-third of the historical warming effect that could have been produced by anthropogenic greenhouse gas emissions alone since the preindustrial era (*25*).

Compared to volcanism, metamorphic devolatilization is an intrinsically slower process. Metamorphic reactions themselves occur over at least hundreds of years even in relatively hot, geologically short-lived contact aureole environments (*26*–*28*). It is likely that a metamorphic sulfur flux occurring over, e.g., ~100 years could drive global cooling [and, perhaps, as Schmidt *et al.* (*19*) suggest, extinction] on a comparable timescale. We therefore undertake exploratory modeling of metamorphic devolatilization around a LIP and the resultant evolution of the climate system. This modeling shows that realistic metamorphic sulfur injection, even coupled with competing greenhouse gas emissions, has the potential to drive catastrophic global cooling of several kelvins sustained over centuries.

**Fig. 2.**
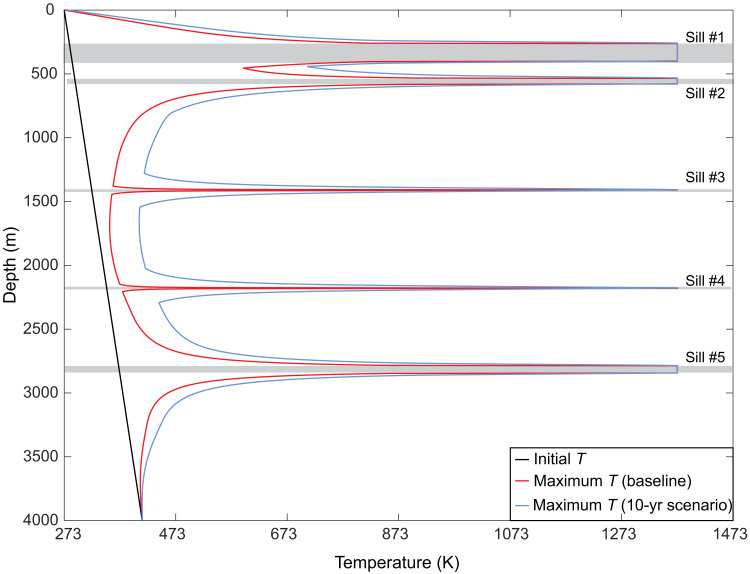
Thermal modeling of intrusion. The initial temperature profile is shown along with the maximum temperature at each depth in the baseline model (red) and the 10-year (yr) intrusion scenario (blue). The sill numbers correspond to their order of intrusion (from earliest to latest).

**Fig. 3.**
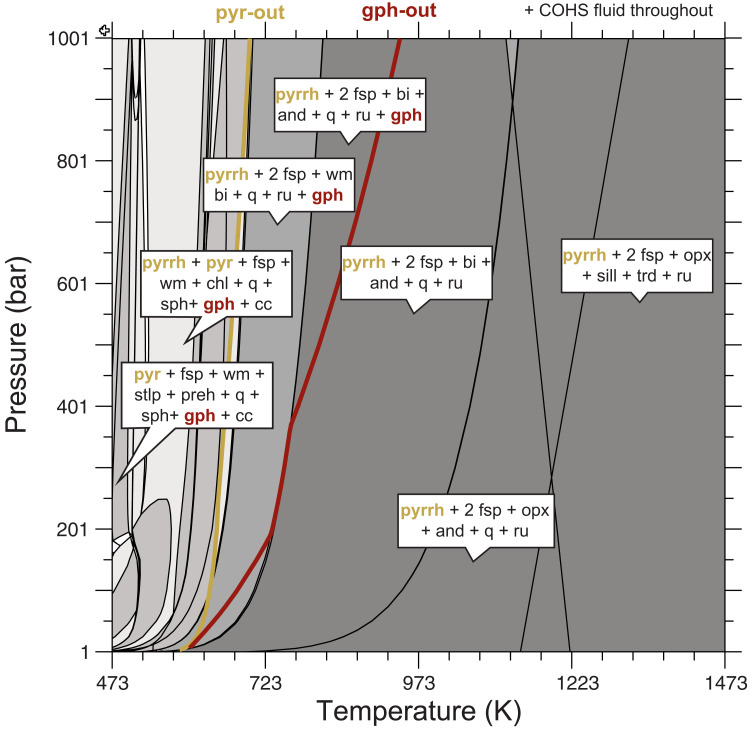
Equilibrium *P*-*T* phase diagram. The *P*-*T* pseudosection calculated in Theriak-Domino is shown with carbon- and sulfur-bearing phases/reactions highlighted.

**Fig. 4.**
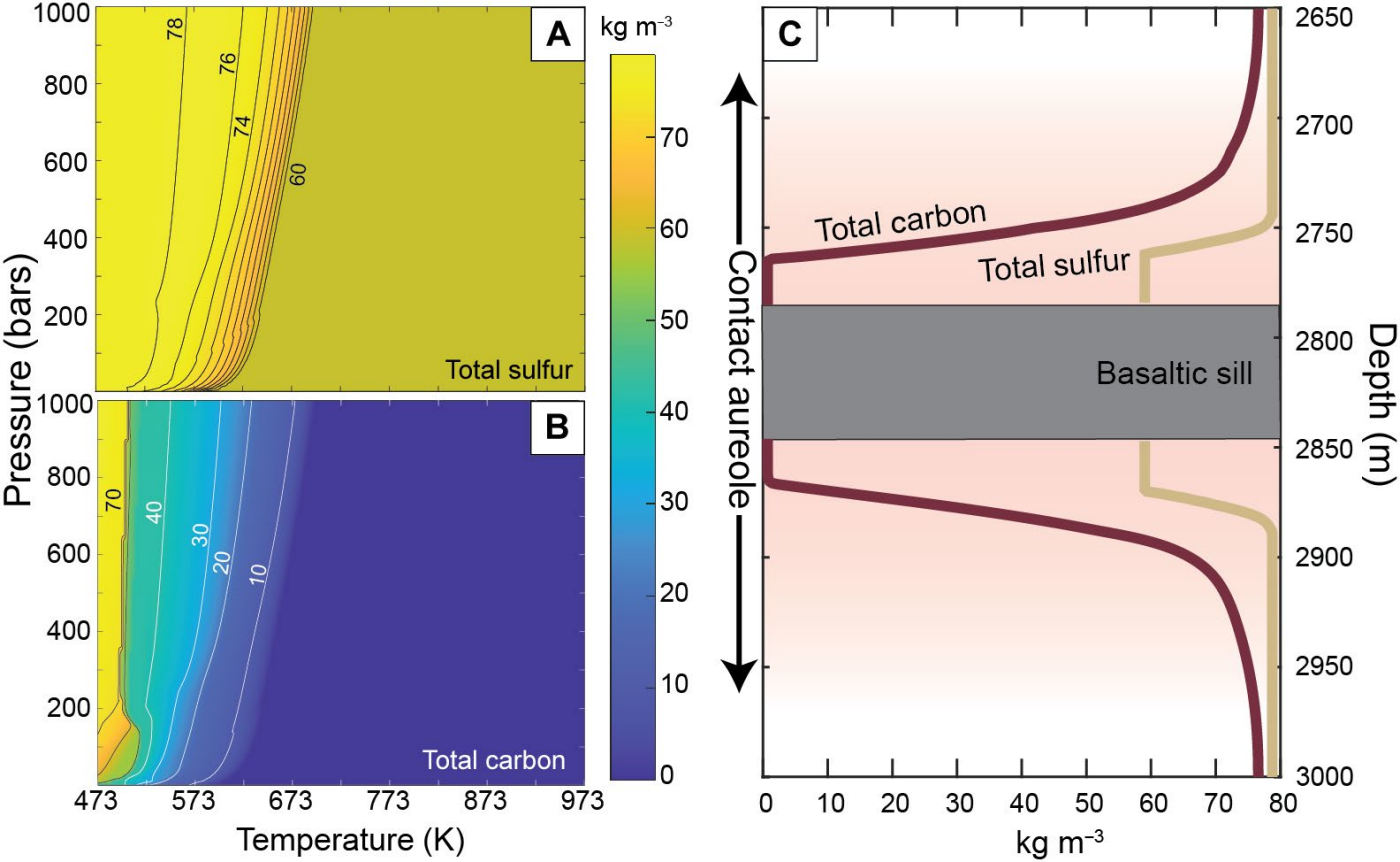
Metamorphic devolatilization. (**A** and **B**) Calculated equilibrium *P*-*T* diagrams showing total sulfur and total carbon, respectively, for a sulfidic black shale composition. At high temperatures, modeling predicts 100% decarbonation and ~25% sulfur release. (**C**) Example contact aureole where the carbon and sulfur contents of the sulfidic black shale country rock decrease adjacent to the intruding basaltic sill.

**Fig. 5.**
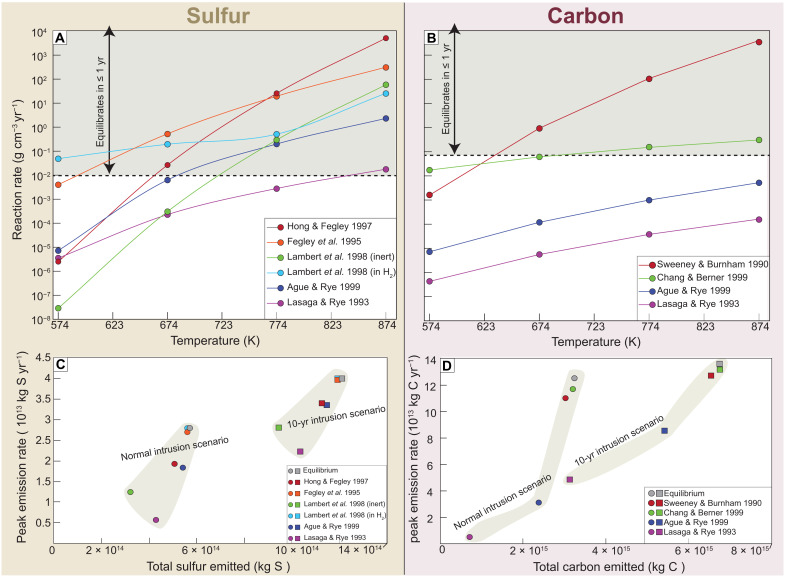
Metamorphic reaction kinetics and their effects on volatile emissions. (**A** and **B**) Literature-based temperature-dependent reaction rates for the sulfur- and carbon-releasing reactions, respectively, on a log scale. At a given temperature, the different reaction rates span many orders of magnitude. Red: (*30*); orange: (*40*); green and blue: (*39*); dark blue: (*31*); purple: (*28*). (**C** and **D**) Peak emission rate and total mass emitted for sulfur and carbon, respectively, as predicted by our thermal-kinetic modeling. Red: (*42*); green: (*41*); dark blue: (*31*); purple: (*28*). Note that results are shown in kg S or C as metamorphic volatiles are not necessarily initially released in the oxidized forms of SO_2_ or CO_2_ (as used in subsequent climate modeling). Emissions assuming instantaneous equilibration are shown with gray symbols, while emissions in an extended 10-year duration intrusion scenario are indicated by squares. For sulfur emission, both the peak and total values increase in the extended intrusion scenario. However, carbon emissions with fast (near equilibrium) kinetic models show a minimal increase in the peak emission rate—despite an approximate doubling of the total emissions—for the 10-year intrusion scenario.

Metamorphic volatile release is estimated as follows (Materials and Methods): A plausible sill geometry from the Central Atlantic Magmatic Province [after (*29*)] is used with a new sill intruding every 1000 years at a temperature of 1374 K. Sills range from 3 to 143 m thick. One-dimensional thermal modeling (Fig. 2) is used to predict the pressure-temperature-time path of intruded sediments, which are assumed to have a sulfidic black shale composition with initial total organic carbon (TOC) of 2.8 wt % and total sulfur (TS) of 2.9 wt %. Thermodynamic modeling (Fig. 3) predicts the equilibrium carbon and sulfur content of the sediment under a range of pressure-temperature (*P*-*T*) conditions (Fig. 4, A and B). Last, a variety of geochemical kinetic parameters are used to estimate the rates of carbon and sulfur release as the heated sediments approach equilibrium (Fig. 5, A and B). In our baseline case, we assume that the sills begin to cool immediately after intrusion, and we use the moderate reaction kinetics in (*30*) and (*31*) for sulfur and carbon liberation, respectively (Fig. 4C). The areal flux resulting from the above is multiplied by 100,000 km^2^ for a total flux to the atmosphere. This area is chosen to represent a conservative overlap between a LIP intrusive area and a shale-bearing basin (Fig. 1). We also consider an alternate intrusion scenario to approximate prolonged magma transport (*32*), where the igneous sill is held at a temperature of 1374 K for 10 years before being allowed to cool (Fig. 2).

The carbon and sulfur fluxes resulting from the metamorphic modeling drive a simple three-box atmosphere/mixed-layer ocean/deep ocean carbon cycle model and two-box mixed-layer ocean/deep ocean climate model emulator to estimate their possible effects (Materials and Methods). The carbon cycle model includes representations of atmosphere-ocean gas exchange and deep ocean overturning tuned to observations (*33*). Radiative forcing from CO_2_ is calculated in the climate model emulator on the basis of the atmospheric concentration values resulting from the carbon cycle model (see Materials and Methods). Radiative forcing from the sulfur fluxes is calculated for both aerosol-radiation interactions (ARIs; direct reflection from sulfate) and aerosol-cloud interactions (ACIs). As our baseline case, we take the emulator as trained on a climate model that has relatively conservative sensitivity to sulfur dioxide emissions [IPSL-CM6-LR (*34*)]. Uncertainty is assessed by comparing results obtained with the other 10 models that released the output needed to fit the ARI and ACI emulation equations (*35*).

## RESULTS

**Fig. 6.**
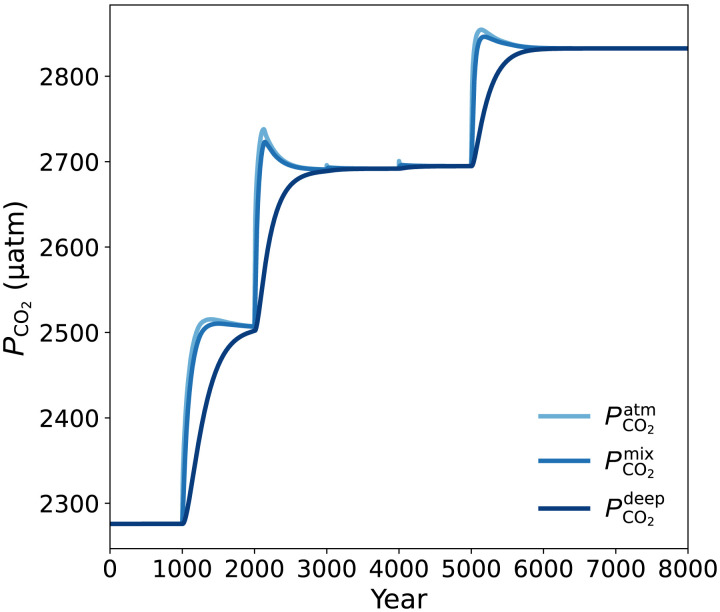
Evolution of CO_2_ concentrations in the atmosphere and mixed-layer and deep oceans. Partial pressures of CO_2_ ($P_{{\text{CO}}_{2}}$) for the atmosphere (atm), mixed-layer ocean (mix), and deep ocean (deep) are shown as light, medium, and dark blue lines, respectively.

The peak emission rate and total emissions for each set of reaction kinetics are shown in Fig. 5 (C and D) for both the baseline and 10-year intrusion scenarios. The reaction rates used span up to seven orders of magnitude at low temperature (Fig. 5, A and B). The resultant peak sulfur fluxes range from ~0.5 × 10^13^ to 4 × 10^13^ kg S year^−1^, equivalent to roughly 1 to 10 Mt. Pinatubo eruptions every day or ~100 to ~500 times the peak historical anthropogenic flux (*36*). The peak carbon fluxes of 0.5 × 10^13^ to 13 × 10^13^ kg C year^−1^ are on the low end of previously estimated thermogenic carbon emissions around LIPs, equivalent to <~20% of modeled peak emissions from, e.g., the North Atlantic Igneous Province (*37*). The total time-integrated carbon emissions are well below existing estimates from the Central Atlantic Magmatic Province in (*29*), primarily due to our smaller area estimate. Despite our relatively conservative models, these sulfur and carbon fluxes and resulting atmospheric concentrations (Fig. 6) are of a sufficient magnitude to drive substantial climate change.

**Fig. 7.**
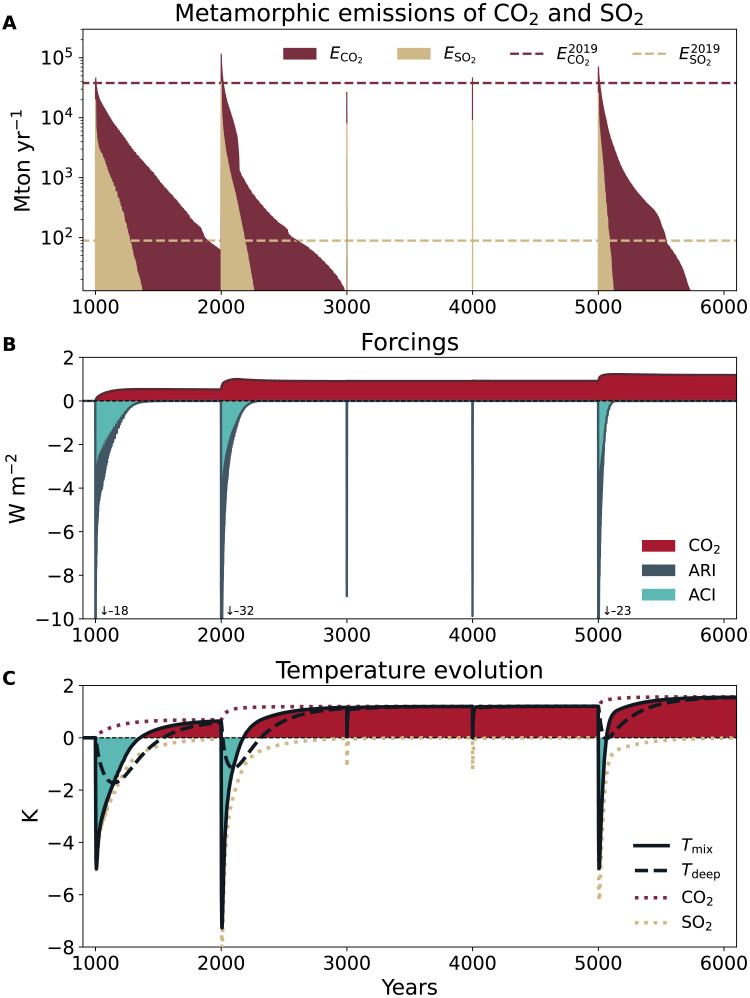
Climate response to thermogenic carbon and sulfur emissions. (**A**) Time series of CO_2_ (garnet shading) using the Ague and Rye (*31*) kinetics and SO_2_ (gold shading) using the Hong and Fegley (*30*) kinetics from the baseline instantaneous emplacement scenario. Dashed lines represent anthropogenic emissions of CO_2_ (garnet) and SO_2_ (gold) from 2019 for reference. (**B**) Resulting CO_2_ (red shading), aerosol-radiation interaction (dark blue shading), and aerosol-cloud interaction (light blue shading) forcing values from the climate model emulator using parameters from the IPSL-CM6A-LR model. (**C**) Resulting temperature anomalies in the mixed-layer ocean (black solid line) and deep ocean (black dashed line) and mixed-layer temperature anomaly contributions from CO_2_ emissions alone (garnet dotted line) and SO_2_ emissions alone (gold dotted line). Red and blue shading indicates periods of net warming and cooling, respectively.

**Fig. 8.**
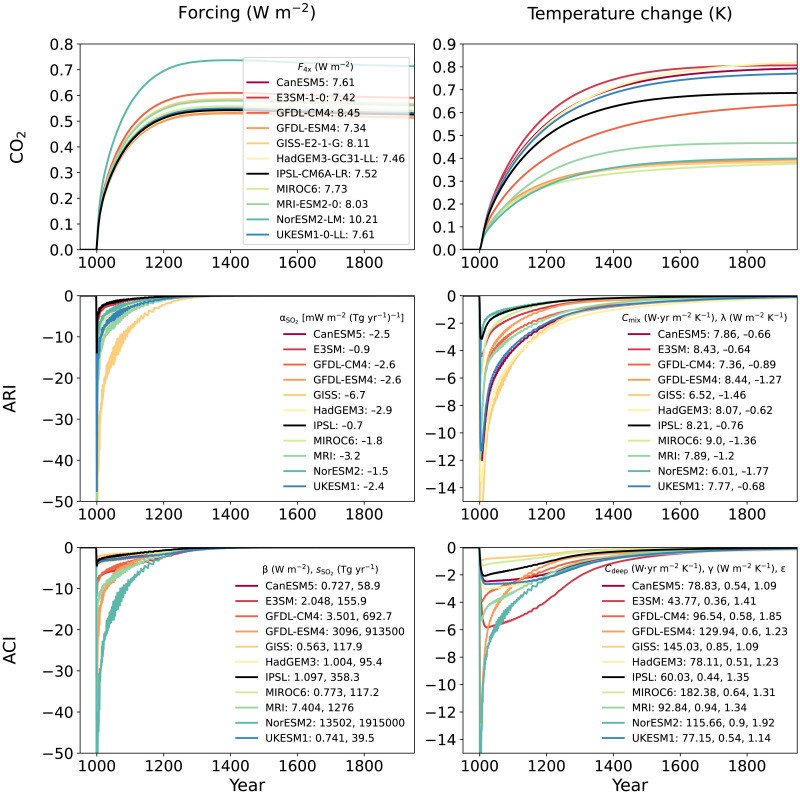
Sensitivity of forcing and temperature response to individual model fittings. Forcing and mixed-layer ocean temperature change (left and right columns, respectively) due to CO_2_ concentrations (top row), aerosol-radiation interactions (middle row), and aerosol-cloud interactions (bottom row) from the simple climate model emulator fit with different coupled models (colored lines). Values are for the baseline case of instantaneous emplacement with Ague and Rye (*31*) kinetics for CO_2_ emissions and Hong and Fegley (*30*) kinetics for SO_2_ emissions. Values of relevant emulator parameters are shown for each model fit in the legends. Only the first sill emplacement is shown for readability.

Figure 7 shows the expected climatic response of our baseline model. The carbon emissions drive a long-term multimillennial warming trend with temperatures elevated by more than +1 K. The sulfur emissions drive more variable, shorter-lived responses depending upon the width of the intruding sill. The largest sill (143 m thick) intruding at 1000 years results in peak cooling of −5 K, with a total cooling duration of about two centuries in the mixed-layer ocean. The deep ocean experiences a smaller magnitude of peak cooling but over a longer duration. Smaller sills (4 and 3 m thick at 3000 and 4000 years, respectively) have very short-lived cooling effects, and they also contribute minimally to the long-term warming trend. Emulator results trained on different models all show qualitatively similar results despite quantitative differences in the balance of heating and cooling (Fig. 8).

**Fig. 9.**
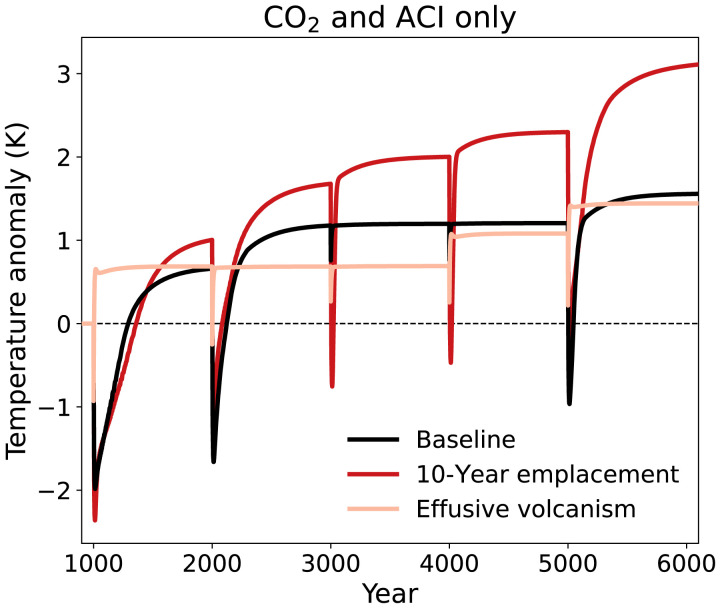
Climate response to thermogenic carbon and sulfur emissions under different sill emplacement or volcanic emission scenarios, excluding aerosol-radiation interactions. Mixed-layer ocean temperature anomalies from the climate model emulator trained on the IPSL-CM6-LR model are shown for the baseline case of instantaneous emplacement (black line), a case of a 10-year emplacement process (red line), and an effusive volcanism–like case of instantaneous emission (pink line). CO_2_ emissions use the Ague and Rye (*31*) kinetics, and SO_2_ emissions use the Hong and Fegley (*30*) kinetics for all cases. The net effect of positive CO_2_ forcing and negative aerosol-cloud interaction forcing is shown; negative forcing from aerosol-radiation interactions is excluded because of unphysical results in the effusive volcanism–like case resulting from severely violated assumptions of linearity between direct aerosol forcing and SO_2_ emissions.

Longer emplacement drives climate changes of greater magnitude but similar longevity. In Fig. 9, the instantaneous-emplacement baseline case is compared with the 10-year emplacement scenario and a scenario with short, pulsed emissions in which a time series emulating effusive volcanism is constructed by compressing the total emissions from any given sill intrusion into a single year. The latter is not intended to replicate realistic volcanism per se but to explore the influence of the tempo of emissions in the troposphere. We exclude ARI here, as the effusive eruption scenario produces unphysically large responses (>30-K cooling) because of the assumption of linearity of ARI forcing with sulfur emissions in the climate model emulator, which very clearly would be violated for the fluxes contemplated here. The total magnitude and duration of the sulfur-driven cooling effect are not overly changed in the 10-year emplacement scenario, but the competing greenhouse effect is substantially magnified by the longer duration of sediment heating. For the effusive volcanism–like scenario, the cooling response is largely restricted to the single year in which low-altitude volcanic-type emissions occur, as expected, and the warming response is still extended over millennia.

## DISCUSSION

The magnitudes of metamorphic carbon and sulfur emissions predicted by our model are substantial. This is consistent with many existing, sophisticated models for thermogenic carbon emission around LIPs (*9*, *29*, *38*). However, the existing literature has not previously included a corresponding estimate for sulfur degassing. While our results incorporate many assumptions and simplifications, they demonstrate that LIP-driven metamorphic sulfur emissions could be of a sufficient rate and magnitude to drive global climate change; this should motivate follow-on work with more sophisticated chemistry-climate models (*19*).

We also demonstrate that the estimated emissions rely heavily on the chemical reaction kinetics at play. The several orders of magnitude of uncertainty in reaction rates translate to substantial differences in the peak emission rate and the total degree of degassing. Some of the reaction rates we use correspond to different reaction mechanisms: For example, the sulfur release kinetics include rates of generic metamorphic reaction (*28*, *31*) and pyrite decomposition in inert gas (*30*, *39*), in CO_2_ (*40*), or in hydrogen gas (H_2_) (*39*). Carbon release includes the same generic metamorphic reaction kinetics along with organic carbon oxidation (*41*) and organic matter maturation (*42*). Given that carbon and sulfur liberation relies on redox reactions, the oxygen fugacity, fluid availability, and presence of other possible redox couples are expected to exert control on the reaction mechanisms and corresponding kinetics.

The climate response predicted by our modeling shows spikes of marked cooling superimposed on a longer-term moderate warming trend. The duration and magnitude of cooling are more sensitive to varying model parameters, but cooling of several kelvins lasting multiple centuries is a robust feature. This sustained cooling results from a single sill intrusion event. Such prolonged cooling from volcanic emissions, even in the stratosphere, is only achievable through a series of fortuitously (or, perhaps, unfortuitously) timed separate volcanic events [e.g., (*43*)]. We suggest that metamorphic sulfur emissions provide a simpler, alternative pathway to achieve centennial-scale cooling. Our selected recurrence interval of 1000 years guarantees that each cooling pulse is isolated in time, but a shorter recurrence interval between sill intrusions [e.g., (*37*) uses ~1 to 10 years in their modeling of North Atlantic Igneous Province sills] would be expected to drive cooling spikes of a greater intensity and duration. A recurrence interval longer than 1000 years would increase the time between cooling events but not their individual durations.

**Fig. 10.**
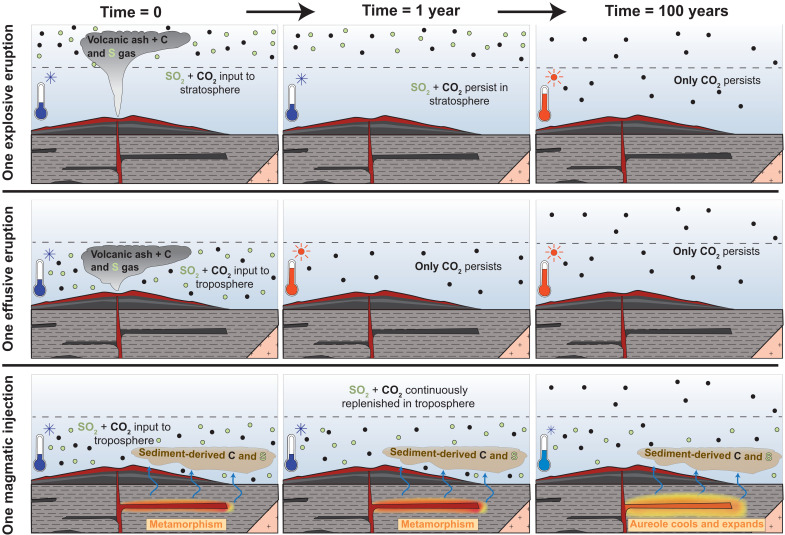
Schematic of the immediate, short-term, and long-term climate effects of carbon and sulfur emissions from an explosive volcanic eruption, an effusive volcanic eruption, or the emplacement of a sill into volatile-rich sedimentary rocks. Explosive volcanic eruptions that reach the stratosphere (top row) can produce strong cooling from stratospheric sulfate aerosols for several years, but only CO_2_ will persist over long time periods. Sulfate from effusive eruptions (middle row) would produce immediate cooling but would be removed from the atmosphere within days to weeks, again leaving only CO_2_ in the atmosphere. In contrast to eruptions, thermogenic sulfur release from a magmatic injection (bottom row) can be sustained over centennial timescales, with a constantly replenished source of sulfate producing long-term cooling.

Our results have potential implications throughout the Earth system. In particular, many of Earth’s mass extinction events may include some component of short-term cooling. As our ability to study these events at high resolution improves, more studies find that climate oscillation (e.g., alternating heating and cooling) is required to drive catastrophic mass extinction. Schmidt *et al.* (*19*) suggest that LIP-derived sulfur emissions would need to be sustained for multiple centuries to cause a biotic crisis. Steinthorsdottir *et al.* (*44*) find evidence for elevated tropospheric sulfur concentrations during the End-Triassic Mass Extinction using SO_2_-induced cuticle damage recorded in fossil plants. Olsen *et al.* (*45*) even argue that transient cold events—as evidenced by abundant lake ice-rafted debris deposits in the Junggar Basin (paleolatitude of 71°N) around the Triassic-Jurassic boundary—contributed to the rise of feather-insulated dinosaurs and pterosaurs and the concurrent decline of the reptilian, noninsulated pseudosuchian archosaurs (e.g., crocodylomorphs). Last, both the End-Triassic and End-Permian Mass Extinction events may be better correlated with the timing of intrusive igneous activity than volcanism, consistent with a metamorphic kill mechanism (*7*, *46*). We suggest that metamorphic sulfur release is a plausible driver of global cooling and other dangerous environmental conditions such as acid rain during ancient extinction intervals. Furthermore, the duration of metamorphic degassing suggested by our modeling is more immediately reconcilable with observed environmental effects than more time-limited volcanic eruptions. We do not mean to imply that volcanic emissions are unimportant but rather that metamorphic sulfur release offers an alternative and complementary explanation for more prolonged global cooling (Fig. 10).

It remains unclear how evidence of centennial scale cooling might appear in the paleoclimate rock record. The cooling spikes we predict in the shallow ocean are shorter than could be seen in the highest-resolution sedimentary records from extinction intervals, while those in the deep ocean may approach detectability. The cooling intervals on the order of 10,000 years, which are observed in the rock record [e.g., (*5*, *47*)], could perhaps result from time averaging of some oscillatory cooling/warming events, but such an interpretation is largely untestable.

One major uncertainty in the translation of emitted SO_2_ to atmospheric SO_4_ concentrations and then to climate forcing is the role of saturation effects from oxidant limitations under such large SO_2_ concentrations. Previous chemistry-climate modeling has shown that the depletion of hydroxyl radical, OH, and other oxidants in flood basalt volcanism can strongly limit how much SO_2_ is ultimately oxidized to form sulfate aerosol. Changes in the oxidative capacity of the atmosphere under sustained (centennial-scale) thermogenic SO_2_ fluxes are a critical area for future research. The unrealistically large ARI values in some models due to assumed linearity with SO_2_ emissions in the climate model emulator are likely to be resolved by accounting for atmospheric oxidation and transport in a more sophisticated manner.

## MATERIALS AND METHODS

### Experimental design

We use sequential models to explore the effect of metamorphic volatile emissions on global climate. First, petrologic thermodynamic modeling predicts the amount of carbon or sulfur a shale composition may contain under given *P*-*T* conditions. Simple thermal modeling of realistic sill geometries generates likely pressure-temperature-time paths of intruded sediments. Coupled kinetic/flux calculations are used to quantify the mass of sulfur and carbon outgassed at the surface as a result of heating. These surface fluxes are passed to a carbon cycle box model and, subsequently, a global energy balance model to estimate climatic effects.

### Thermodynamic modeling of metamorphism

Thermodynamic modeling was performed in the system Na_2_O + CaO + K_2_O + FeO + MgO + Al_2_O_3_ + SiO_2_ + H_2_O + TiO_2_ + C + S + O in the Theriak-Domino software package (*48*) with the internally consistent thermodynamic database of Holland and Powell (*49*) and compatible activity models (table S1). The bulk chemical composition of the country rock was chosen as an organic and sulfide-rich Silurian shale sample AGC-12 from (*50*), which reports bulk rock compositions with weight % oxides, weight % loss on ignition (LOI), TOC, and TS. All iron was assumed to be Fe^2+^ (a reasonable assumption in an evidently highly reduced rock), and the measured LOI was corrected accordingly. After correcting LOI for the Fe^3+^-to-Fe^2+^ conversion, we calculated weight % water (H_2_O) by *wt % H*_*2*_*O* = *wt % LOI*_corrected_ − *TOC* − *TS*. Calcium oxide (CaO) was adjusted for any phosphate present, assuming apatite stoichiometry such that *wt % CaO*_corrected_ = *wt % CaO*_measured_ − 1.331 × (*wt % P*_*2*_*O*_*5*_). In converting wt % oxides into moles, the oxygen required for charge balance was calculated, assuming that all C was present as graphite (i.e., C^o^) and all sulfur was in pyrite (FeS_2_).

A *P*-*T* phase diagram (pseudosection) was calculated in a closed chemical system (Fig. 3). Individual calculations at a pressure of 100 bars and temperatures from 373 to 1473 K were performed to track changing mineral modes and identify relevant reactions. For all subsequent calculations, we only consider sulfur loss in the temperature range of ~500 to ~700 K where sulfur is degassed via the reaction

$$\begin{array}{c} {\text{FeS}}_{2\left(\text{pyrite}\right)}+0.5\;C_{\left(\text{graphite}\right)}+H_{2}O_{\left(\text{fluid}\right)}=0.91\;H_{2}S_{\left(\text{fluid}\right)}+\\ 0.5\;{\text{CO}}_{2\left(\text{fluid}\right)}+0.1\;H_{2\left(\text{fluid}\right)}+0.7008\;{\text{FeS}}_{\left(\text{trov}\right)}+0.4408\;{\text{Fe}}_{0.88}S_{\left(\text{troilite}\right)} \end{array}$$ (1)

By this temperature, only about half of the mineral-bound sulfur is hosted in pyrite (the other half is in pyrrhotite). Therefore, the fraction of the rock’s initial sulfur that is released by this reaction is ~25% (see Fig. 4B). *P*-*T* modeling predicts that sulfur degassing will continue up to ~32% loss by ~1300 K as pyrrhotite becomes more Fe-rich, but we ignore this contribution because such temperatures are unlikely to be sustained in a contact aureole. Furthermore, our thermodynamic model does not consider the melting processes that may predominate in this higher temperature range.

Carbon loss begins at lower temperatures (~300 K), and the rocks achieve 100% equilibrium decarbonation by about 700 K (see Fig. 4C). For the purpose of subsequent kinetic calculations, we assume that carbon is devolatilized by the reaction

$$C_{\left(\text{graphite}\right)}+H_{2}O_{\left(\text{fluid}\right)}=0.5\;{\text{CH}}_{4\left(\text{fluid}\right)}+0.5\;{\text{CO}}_{2\left(\text{fluid}\right)}$$ (2)

### Reaction kinetics

We consider a wide range of possible metamorphic reaction kinetics for the liberation of both sulfur and carbon. We can describe the rate for the reaction of interest in some arbitrary volume according to

$$\frac{\partial m_{i}}{\partial t}=k\nu_{i}A{|\Delta G_{r}|}^{n}$$ (3)

where $\frac{\partial m_{i}}{\partial t}$ is the change in moles of species *i* with time, *k* is the specific reaction rate, ν*_i_* is the stoichiometric coefficient of *i* in the reaction (defined as negative for reactants and positive for products), *A* is the reactive surface area, Δ*G*_r_ is the Gibbs free energy change of the reaction, and *n* is the reaction order. The value of *k* is in turn dependent on some Arrhenius relation

$$k=k_{o}\text{exp}\left[\frac{-E_{a}}{R}\left(\frac{1}{T}-\frac{1}{T_{o}}\right)\right]$$ (4)

where *k*_o_ is the reaction rate constant at some reference temperature *T*_o_, *E*_a_ is the activation energy, and *R* is the gas constant.

#### Sulfur

There are no published studies specifically on the rate of Eq. 1 that is indicated by our thermodynamic modeling. We thus turn to a variety of similar reactions with kinetic data from the literature.

##### Pyrite decomposition

Many experimental studies estimate the rate of decomposition of pyrite to form pyrrhotite and elemental sulfur, S_2_ gas, or hydrogen sulfide (H_2_S) gas at elevated temperatures and low/moderate pressure. The reaction rate is strongly dependent on temperature and redox conditions but is likely a zero-order reaction [i.e., not dependent on the amount of reactant present (*51*)]. Lambert and others (*39*) estimate pyrite decomposition rates in both inert atmospheres and with H_2_ present. Hong and Fegley (*30*) consider decomposition in a variety of atmospheres including CO_2_ and both oxidizing and reducing conditions, while Fegley and others (*40*) recreate temperature and high CO_2_ atmospheric conditions similar to the Venusian surface. Activation energies range from 156 kJ mol^−1^ in an oxidizing environment to 297 kJ mol^−1^ in inert gas, and resultant reaction rates span up to seven orders of magnitude at ~600 K (see Fig. 5A)

##### Generic metamorphic reactions

We also apply kinetics relevant to generic metamorphic reaction after Ague and Rye (*31*) and Lasaga and Rye (*28*). These reaction rates are generally slower with lower activation energies (set to 83.7 kJ mol^−1^) but smaller rate constants. In addition, these are formulated with a reaction order of 2.68 or 1 for Ague and Rye (*31*) and Lasaga and Rye (*28*), respectively. These therefore rely on Δ*G*_r_ (the Gibbs free energy change of the reaction), which depends upon phase activities according to

$$\Delta G_{r}=\Delta {G^{*}}_{r}+\mathrm{RT}\text{ln}\left(Q_{r}\right)={{\Delta G}^{*}}_{r}+\mathrm{RT}\text{ln}\left(\prod {a_{j}}^{\nu_{j}}\right)$$ (5)

where $\Delta {G^{*}}_{r}$ is the Gibbs free energy change of the reaction at the standard state (e.g., pure phases at the *P* and *T* of interest), *Q*_r_ is the activity product (analogous to an equilibrium constant without the assumption of equilibrium), *a_j_* is the chemical activity of the *j*th phase, and ν*_j_* is its stoichiometric coefficient. For sulfur liberation, we assume Eq. 1 above. The value of $\Delta {G^{*}}_{r}$ can be calculated in Theriak-Domino program Thalia (fig. S1A). In nature, the activity product (*Q*_r_) will vary as a function of reaction progress, pressure, and temperature. For simplicity, we assume a constant value of *Q*_r_ = 0.00285. This corresponds to the predicted equilibrium product/reactant activities at 593 K and 100 bars, with *a*_H2O_ = 0.648, *a*_H2S_ = 0.012, *a*_CO2_ = 0.193, *a*_H2_ *=* 0.0038, *a*_troilite_ = 0.298, and *a*_trov_ = 0.606. Note that these *P*-*T* conditions are chosen to represent the biggest “pulse” in sulfur release predicted by our equilibrium thermodynamic modeling. These assumptions introduce some error, but it is dwarfed by the uncertainty in other kinetic parameters.

#### Carbon

Our thermodynamic modeling considers only the phase graphite for reduced carbon mineralogy. In nature, carbon is likely present in more complex organic material, and we consider a few sources of reaction kinetics for both graphite and organic carbon (sensu stricto) from the literature. Chang and Berner (*41*) present estimated kinetic parameters for the oxidation of sedimentary organic matter [CH_2_O (formaldehyde)] into CO_2_ gas with zeroth order kinetics and an activation energy of 30 kJ mol^−1^. The Easy%Ro model (*42*) estimates the maturation of organic matter via 20 separate reactions. We adopt a simplified representation of these kinetics with an average activation energy of 222 kJ mol^−1^ from a distribution with a standard deviation of 49 kJ mol^−1^ (1σ). We also adopt an average stoichiometric value of C in fluid of *ν*_c_ = 0.425 (that is, 0.425 moles of C is degassed per reactant mole of organic C). Again, these are simplifications relative to the full Easy%Ro model, but they become insignificant in cross-model comparison.

Last, we also consider generic metamorphic reaction kinetics, as described above. For the release of carbon via Eq. 2, $\Delta {G^{*}}_{r}$ is calculated at each *P*-*T* pair in Theriak-Domino (fig. S1B), and *Q*_r_ is fixed at 0.017 (with *a*_H2O_ = 0.997, *a*_CH4_ = 0.159, and *a*_CO2_ = 0.002) corresponding to equilibrium conditions at 423 K and 100 bars, the conditions of the greatest carbon degassing.

### Thermal modeling

The geometry of sills is taken from borehole OAST after (*29*). The initial linear geotherm is set with a temperature of 273 K at the surface and 343 K at a depth of 2000 m, also after (*29*). One-dimensional finite-difference modeling with a second derivative scheme and Dirichlet boundary conditions at depths of 0 and 4000 m is used to predict the temperature evolution of sediments after sill intrusion (Fig. 2). A single thermal diffusion constant of 10^−6^ m^2^ s^−1^ is used throughout.

### Flux calculation

The results of one-dimensional thermal modeling are converted to pressure-temperature-time paths using a hydrostatic gradient of 98 bars/km. The rock density was taken as 2700 kg m^−3^. Results reported herein used a time step of 1 year for flux calculations, but sensitivity analysis with time steps as short as 0.05 year shows no substantial differences (fig. S2). At each time step, the equilibrium C and S content of the sediment under its current *P*-*T* condition is extracted from thermodynamic models. No readdition of C or S is allowed. If the predicted equilibrium C or S content is below the content from the previous time step, the sediment proceeds toward equilibrium according to the assigned reaction kinetics. In the “equilibrium” scenario, the sediment achieves equilibrium at each time step with no kinetic limitation. Any released C or S is assumed to fully escape the rock column within the same 1-year time step, a reasonable assumption within the hydrostatic realm where flow rates are generally above 5000 m year^−1^. Degassed C and S are assumed to be oxidized into forms CO_2_ and SO_2_, respectively, before consideration in subsequent carbon cycle or atmospheric modeling.

#### Simple carbon cycle model

CO_2_ is long-lived in the atmosphere; it is only fully removed by geological sequestration over many thousands of years. It does enter the shallow ocean on annual timescales however, and then the deep ocean over centuries. We treat the Earth system as being composed of three “boxes”: an atmosphere, a mixed-layer ocean (in contact with the atmosphere), and a deep ocean.

#### Atmosphere

CO_2_ is added to the atmosphere through emissions from the metamorphic source and removed through a sink via the surface ocean. We do not consider the terrestrial carbon cycle here. The rate of exchange of CO_2_ between the atmosphere and the ocean is governed by a transfer coefficient (*33*) that is the product of gas transfer velocity (κ) related to wind speed, temperature, and salinity and the solubility of CO_2_ in seawater (α). We adopt a representative κα product value of 0.05 year^−1^. Changes in atmospheric CO_2_ concentrations are calculated as

$$\frac{d{P\text{co}}_{2}^{\text{atm}}}{\mathrm{dt}}=E_{CO_{2}}-\kappa \alpha \left({P\text{co}}_{2}^{\text{atm}}-{P\text{co}}_{2}^{\text{mix}}\right)$$

where ${P\text{co}}_{2}^{\text{atm}}$ and ${P\text{co}}_{2}^{\text{mix}}$ are the partial pressures of CO_2_ in the atmosphere and mixed-layer ocean, respectively, and $E_{CO_{2}}$ is the emission rate of CO_2_ calculated above.

#### Mixed-layer ocean

CO_2_ is added to the mixed-layer ocean through exchange with the atmosphere, as above, and removed via mixing with the deep ocean. For the latter, we define a mixing parameter ζ, the inverse of which gives the characteristic ocean overturning timescale. We adopt a representative ζ value of 0.004 year^−1^. Changes in ${P\text{co}}_{2}^{\text{mix}}$ are then calculated as

$$\frac{d{P\text{co}}_{2}^{\text{mix}}}{\mathrm{dt}}=\kappa \alpha \left({P\text{co}}_{2}^{\text{atm}}-{P\text{co}}_{2}^{\text{mix}}\right)-\zeta \left({P\text{co}}_{2}^{\text{mix}}-{P\text{co}}_{2}^{\text{deep}}\right)$$

where ${P\text{co}}_{2}^{\text{deep}}$ is the partial pressure of CO_2_ in the deep ocean.

#### Deep ocean

CO_2_ is added to the deep ocean through transport from the mixed-layer ocean. Ultimately, carbon will be removed through sedimentation and burial, but this process is negligibly slow given an analysis period of only a few millennia. Changes in ${P\text{co}}_{2}^{\text{deep}}$ are thus calculated as

$$\frac{d{P\text{co}}_{2}^{\text{deep}}}{\mathrm{dt}}=\zeta \left({P\text{co}}_{2}^{\text{mix}}-{P\text{co}}_{2}^{\text{deep}}\right)$$

As our initial condition, we assume that all of the carbon reservoirs are in equilibrium with an initial atmospheric concentration ($p_{0}$) of 1500 ppm (parts per million) by volume (*52*). The evolution of CO_2_ concentrations in the atmosphere and mixed-layer and deep oceans is presented in Fig. 6.

### Climate model emulator

To understand how the thermogenic CO_2_ and SO_2_ emissions would affect global climate over long time periods, we adapt the simple energy balance model, trained on much more complex global climate models, from Smith *et al.* (*35*). For the base case shown in Figs. 7 and 9, we only use parameters fit using the IPSL-CM6A-LR climate model (*34*), which had relatively conservative responses to both CO_2_ and SO_2_ compared to the other models analyzed. Figure 8 shows the sensitivity of our results to the choice of climate model, focusing on the first sill emplacement for clarity.

#### CO_2_ forcing

The effective radiative forcing from changes in CO_2_ ($F_{{\text{CO}}_{2}}$) (*53*) in our simulations is estimated using values derived from abrupt-4xCO2 experiments (instantaneously quadrupling atmospheric CO_2_ concentration) from each model ($F_{4x}$) (*35*)

$$F_{CO_{2}}=\frac{F_{4x}}{\text{ln}4}\text{ln}\left(\frac{{P\text{co}}_{2}^{\text{atm}}}{p_{0}}\right)$$

#### Direct aerosol forcing

The direct aerosol radiative effect refers to how scattering (reflection) and absorption of light by aerosol particles influence Earth’s energy budget. We parameterize the effective radiative forcing due to aerosol-radiation interactions (direct effect and changes in clouds caused by related changes in atmospheric temperature profiles), $F_{\text{ARI}}$, as

$$F_{\text{ARI}}=\alpha_{SO_{2}}E_{SO_{2}}$$

where $\alpha_{SO_{2}}$ is a scaling coefficient fit from global climate model simulations of historical and preindustrial aerosol emissions (*35*). Because this formulation is linear, it is unlikely to hold for $E_{SO_{2}}$ values much larger than those experienced historically and those that the emulator was trained on.

#### Indirect aerosol forcing

The aerosol indirect radiative effect refers to how changes in clouds—caused by the role of aerosol particles in nucleating cloud droplets—influence Earth’s energy budget. For the same volume of liquid water in a cloud, spreading the water over a larger number of small droplets maximizes the effective surface area (Twomey effect) (*54*). The total volume of liquid can also change because of cloud adjustments to the Twomey effect, such as a reduction in precipitation (extra cooling) (*55*) or an increase in evaporation (offsetting warming) (*56*). We parameterize the effective radiative forcing due to aerosol-cloud interactions (Twomey effect and adjustments), $F_{\text{ACI}}$, as

$$F_{\text{ACI}}=-\mathrm{\beta ln}\left(1+\frac{E_{SO_{2}}}{s_{SO_{2}}}\right)$$

where β and $s_{SO_{2}}$ are scaling coefficients fit from global climate model simulations of historical and preindustrial aerosol emissions (*35*). Models with larger $s_{SO_{2}}$ values have more linear responses and are thus also unlikely to be reliable for large $E_{SO_{2}}$.

#### Temperature response

Temperature evolution follows a simple two-box model of fast (surface ocean) and slow (deep ocean) temperature responses to radiative forcing (*57*). $T_{\text{mix}}$ and $T_{\text{deep}}$ represent temperature anomalies for the mixed-layer and deep oceans, respectively, from an initial equilibrium state. The mixed-layer temperature responds to top-of-atmosphere radiative forcing F (radiative imbalance before any temperature response, which can be any combination of $F_{{\text{CO}}_{2}}$, $F_{\text{ARI}}$, and $F_{\text{ACI}}$). As the temperature changes, the top-of-atmosphere imbalance is resolved at a rate given by the climate feedback parameter λ. There is also heat exchange between the mixed-layer and deep oceans, occurring at a rate given by the product of the efficacy of deep ocean heat uptake ϵ and a heat transport term γ. The heat capacities of the mixed-layer and deep oceans are given by $C_{\text{mix}}$ and $C_{\text{deep}}$, respectively. Temperatures evolve as

$$C_{\text{mix}}\frac{dT_{\text{mix}}}{\mathrm{dt}}=F+\lambda T_{\text{mix}}-\epsilon \gamma \left(T_{\text{mix}}-T_{\text{deep}}\right)$$

$$C_{\text{deep}}\frac{dT_{\text{deep}}}{\mathrm{dt}}=\gamma \left(T_{\text{mix}}-T_{\text{deep}}\right)$$

Fitted parameter values for each model are provided in Fig. 8 and can also be found in table 3 and table S1 in the work of Smith *et al.* (*35*).

## Supplementary Materials

### This PDF file includes:

Figs. S1 and S2 Table S1 References
