# Supplementary material for: Metamorphic sulfur release as a driver of sustained cooling and mass extinction
Source: Sci Adv. 2026 Jul 8;12(28):eaee2277. doi: 10.1126/sciadv.aee2277 (PMC13344362; doi:10.1126/sciadv.aee2277)
Supplement: Supplementary file 1 — Figs. S1 and S2 Table S1 References [file sciadv.aee2277_sm.pdf]

Science Advances
AAAS

Supplementary Materials for

Metamorphic sulfur release as a driver of sustained cooling and mass extinction

Emily M. Stewart *et al.*

Corresponding author: Emily M. Stewart, emstewart@fsu.edu; Michael S. Diamond, msdiamond@fsu.edu

Sci. Adv. **12**, eaee2277 (2026)
DOI: 10.1126/sciadv.aee2277

This PDF file includes:

Figs. S1 and S2
Table S1
References

Fig. S1.

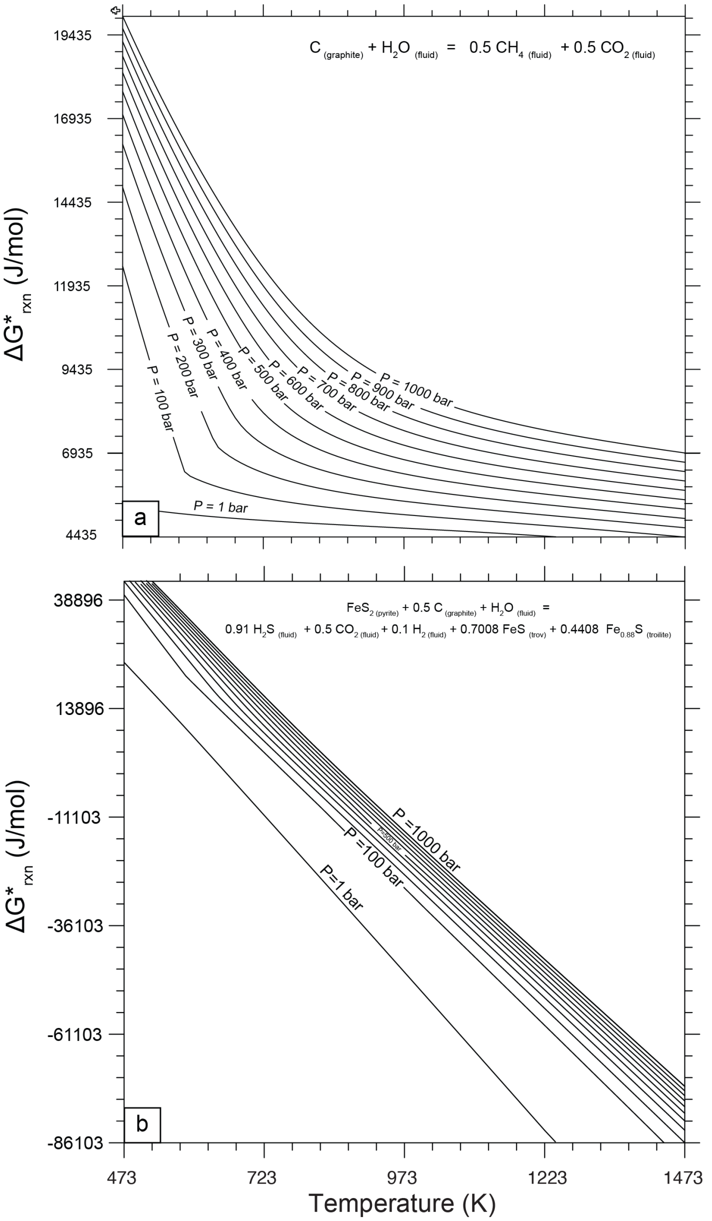

The Gibbs free energy change at standard state. Standard state represents unit reactant/product activities at the P and T of interest. Shown for (a) the carbon releasing reaction and (b) the sulfur releasing reaction as generated by Thalia (*48*). Inclusion of the activity product as in equation 5 will drive both reactions forward.

Fig. S2.

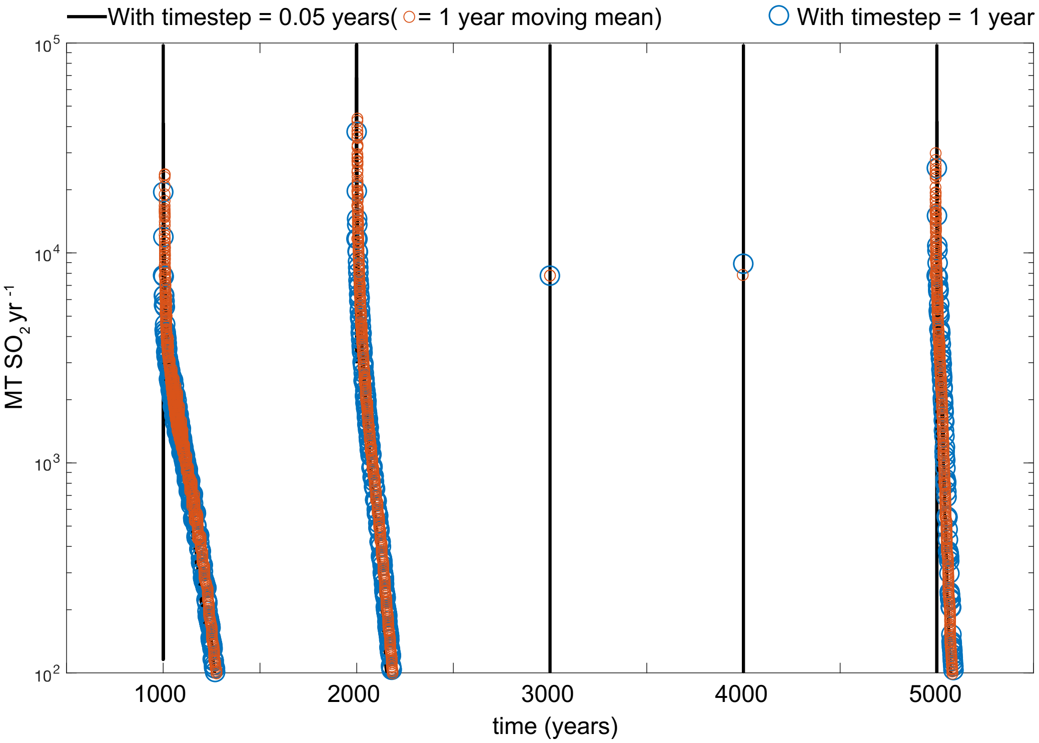

The effect of flux timestep. The SO_2 flux for our baseline case is shown with a shortened timestep of 0.05 years (black line) and one year (blue circles). The two models are in good agreement. The higher values in the 0.05-year model are only present over a few short timesteps, and averaging over one year (orange circles) brings these peak values back in line with the results from timestep=1 year.

Table S1.

Reference	activity model(s)
Holland & Powell 2011 (*49)*	Olivine, Stilpnomelane, Minnesotaite, Prehnite, Pumpellyite, Epidote, Magnetite
Evan *et al.* (*61)*	Pyrrhotite, sulfur species in COHS fluid
Holland & Powell 1998 (*62*)	Dolomite-Ankerite, Magnesite-Siderite
Holland & Powell 2022 (*63)*	Feldspar
White *et al.* 2014 (*64)*	Garnet, Margarite, White Mica, Biotite, Orthopyroxene, Staurolite, Chlorite, Ilmenite
Green et al. 2016 (*65*)	Amphibole, Clinopyroxene
Holland & Powell 2003 (*66)*	COHS fluid (COH species)

The activity models used in thermodynamic modeling in the Theriak-Domino software package are given above.

REFERENCES

1. P. Wignall, The link between large igneous province eruptions and mass extinctions. *Elements* **1**, 293–297 (2005).

2. D. P. Bond, S. E. Grasby, On the causes of mass extinctions. *Palaeogeogr. Palaeoclimatol. Palaeoecol.* **478**, 3–29 (2017).

3. J. Kasbohm, B. Schoene, S. Burgess, "Radiometric constraints on the timing, tempo, and effects of large igneous province emplacement, in *Large Igneous Provinces: A Driver of Global Environmental and Biotic Changes* (Wiley, 2021), pp. 27–82.

4. S. E. Bryan, R. E. Ernst, Revised definition of large igneous provinces (LIPs). *Earth Sci. Rev.* **86**, 175–202 (2008).

5. N. P. Kozik, S. A. Young, S. M. Newby, M. Liu, D. Chen, E. U. Hammarlund, D. P. Bond, T. R. Them, J. D. Owens, Rapid marine oxygen variability: Driver of the Late Ordovician mass extinction. *Sci. Adv.* **8**, eabn8345 (2022).

6. D. V. Kent, P. E. Olsen, H. Wang, M. F. Schaller, M. Et-Touhami, Correlation of sub-centennial-scale pulses of initial Central Atlantic Magmatic Province lavas and the end-Triassic extinctions. *Proc. Natl. Acad. Sci. U. S. A* **121**, e2415486121 (2024).

7. S. D. Burgess, B. A. Black, The anatomy and lethality of the Siberian traps large igneous province. *Annu. Rev. Earth Planet. Sci.* **53**, 567–594 (2025).

8. B. A. Black, R. R. Neely, J.-F. Lamarque, L. T. Elkins-Tanton, J. T. Kiehl, C. A. Shields, M. J. Mills, C. Bardeen, Systemic swings in end-Permian climate from Siberian Traps carbon and sulfur outgassing. *Nat. Geosci.* **11**, 949–954 (2018).

9. I. Aarnes, H. Svensen, J. A. Connolly, Y. Y. Podladchikov, How contact metamorphism can trigger global climate changes: Modeling gas generation around igneous sills in sedimentary basins. *Geochim. Cosmochim. Acta* **74**, 7179–7195 (2010).

10. C. Ganino, N. T. Arndt, Climate changes caused by degassing of sediments during the emplacement of large igneous provinces. *Geology* **37**, 323–326 (2009).

11. H. Svensen, S. Planke, A. Malthe-Sørenssen, B. Jamtveit, R. Myklebust, T. Rasmussen Eidem, S. S. Rey, Release of methane from a volcanic basin as a mechanism for initial Eocene global warming. *Nature* **429**, 542–545 (2004).

12. H. Svensen, S. Planke, A. G. Polozov, N. Schmidbauer, F. Corfu, Y. Y. Podladchikov, B. Jamtveit, Siberian gas venting and the end-Permian environmental crisis. *Earth Planet. Sci. Lett.* **277**, 490–500 (2009).

13. M. T. Jones, D. A. Jerram, H. H. Svensen, C. Grove, The effects of large igneous provinces on the global carbon and sulphur cycles. *Palaeogeogr. Palaeoclimatol. Palaeoecol.* **441**, 4–21 (2016).

14. C. Yallup, M. Edmonds, A. V. Turchyn, Sulfur degassing due to contact metamorphism during flood basalt eruptions. *Geochim. Cosmochim. Acta* **120**, 263–279 (2013).

15. F. M. Deegan, J. H. Bédard, S. E. Grasby, K. Dewing, H. Geiger, V. Misiti, M. Capriolo, S. Callegaro, H. H. Svensen, C. Yakymchuk, Magma–shale interaction in large igneous provinces: Implications for climate warming and sulfide genesis. *J. Petrol.* **63**, egac094 (2022).

16. M. Capriolo, B. J. W. Mills, R. J. Newton, J. Dal Corso, A. M. Dunhill, P. B. Wignall, A. Marzoli, Anthropogenic-scale CO_2 degassing from the Central Atlantic Magmatic Province as a driver of the end-Triassic mass extinction. *Global Planet. Change* **209**, 103731 (2022).

17. L. S. Glaze, S. Self, A. Schmidt, S. J. Hunter, Assessing eruption column height in ancient flood basalt eruptions. *Earth Planet. Sci. Lett.* **457**, 263–270 (2017).

18. S. Callegaro, D. R. Baker, A. De Min, A. Marzoli, K. Geraki, H. Bertrand, C. Viti, F. Nestola, Microanalyses link sulfur from large igneous provinces and Mesozoic mass extinctions. *Geology* **42**, 895–898 (2014).

19. A. Schmidt, R. A. Skeffington, T. Thordarson, S. Self, P. M. Forster, A. Rap, A. Ridgwell, D. Fowler, M. Wilson, G. W. Mann, P. B. Wignall, K. S. Carslaw, Selective environmental stress from sulphur emitted by continental flood basalt eruptions. *Nat. Geosci.* **9**, 77–82 (2016).

20. M. S. Diamond, H. M. Director, R. Eastman, A. Possner, R. Wood, Substantial cloud brightening from shipping in subtropical low clouds. *AGU Adv.* **1**, e2019AV000111 (2020).

21. A. Gettelman, M. W. Christensen, M. S. Diamond, E. Gryspeerdt, P. Manshausen, P. Stier, D. Watson-Parris, M. Yang, M. Yoshioka, T. Yuan, Has reducing ship emissions brought forward global warming? *Geophys. Res. Lett.* **51**, e2024GL109077 (2024).

22. T. Yuan, H. Song, L. Oreopoulos, R. Wood, H. Bian, K. Breen, M. Chin, H. Yu, D. Barahona, K. Meyer, S. Platnick, Abrupt reduction in shipping emission as an inadvertent geoengineering termination shock produces substantial radiative warming. *Commun. Earth Environ.* **5**, 281 (2024).

23. Y. Chen, J. Haywood, Y. Wang, F. Malavelle, G. Jordan, A. Peace, D. G. Partridge, N. Cho, L. Oreopoulos, D. Grosvenor, P. Field, R. P. Allan, U. Lohmann, Substantial cooling effect from aerosol-induced increase in tropical marine cloud cover. *Nat. Geosci.* **17**, 404–410 (2024).

24. F. F. Malavelle, J. M. Haywood, A. Jones, A. Gettelman, L. Clarisse, S. Bauduin, R. P. Allan, I. H. H. Karset, J. E. Kristjansson, L. Oreopoulos, N. Cho, D. Lee, N. Bellouin, O. Boucher, D. P. Grosvenor, K. S. Carslaw, S. Dhomse, G. W. Mann, A. Schmidt, H. Coe, M. E. Hartley, M. Dalvi, A. A. Hill, B. T. Johnson, C. E. Johnson, J. R. Knight, F. M. O'Connor, D. G. Partridge, P. Stier, G. Myhre, S. Platnick, G. L. Stephens, H. Takahashi, T. Thordarson, Strong constraints on aerosol-cloud interactions from volcanic eruptions. *Nature* **546**, 485–491 (2017).

25. P. M. Forster, T. Storelvmo, K. Armour, W. Collins, J.-L. Dufresne, D. Frame, D.J. Lunt, T. Mauritsen, M.D. Palmer, M. Watanabe, M. Wild, H. Zhang, "The Earth's energy budget, climate feedbacks, and climate sensitivity," in *Climate Change 2021: The Physical Science Basis. Contribution of Working Group I to the Sixth Assessment Report of the Intergovernmental Panel on Climate Change* (Cambridge Univ. Press, 2021), chap. 7, pp. 923–1054.

26. A. Skelton, The effect of metamorphic fluid flow on the nucleation and growth of garnets from Troms, North Norway. *J. Metam. Geol.* **15**, 85–92 (1997).

27. A. Skelton, M. Bickle, C. Graham, Fluid-flux and reaction rate from advective-diffusive carbonation of mafic sill margins in the Dalradian, southwest Scottish Highlands. *Earth Planet. Sci. Lett.* **146**, 527–539 (1997).

28. A. C. Lasaga, D. M. Rye, Fluid flow and chemical reaction kinetics in metamorphic systems. *Am. J. Sci.* **293**, 361–404 (1993).

29. T. H. Heimdal, H. H. Svensen, J. Ramezani, K. Iyer, E. Pereira, R. Rodrigues, M. T. Jones, S. Callegaro, Large-scale sill emplacement in Brazil as a trigger for the end-Triassic crisis. *Sci. Rep.* **8**, 141 (2018).

30. Y. Hong, B. Fegley Jr., The kinetics and mechanism of pyrite thermal decomposition. *Ber. Bunsen. Phys. Chem* **101**, 1870–1881 (1997).

31. J. J. Ague, D. M. Rye, Simple models of CO_2 release from metacarbonates with implications for interpretation of directions and magnitudes of fluid flow in the deep crust. *J. Petrol.* **40**, 1443–1462 (1999).

32. J. Biasi, L. Karlstrom, Timescales of magma transport in the Columbia River flood basalts, determined by paleomagnetic data. *Earth Planet. Sci. Lett.* **576**, 117169 (2021).

33. T. Takahashi, S. C. Sutherland, R. Wanninkhof, C. Sweeney, R. A. Feely, D. W. Chipman, B. Hales, G. Friederich, F. Chavez, C. Sabine, A. Watson, D. C. E. Bakker, U. Schuster, N. Metzl, H. Yoshikawa-Inoue, M. Ishii, T. Midorikawa, Y. Nojiri, A. Körtzinger, T. Steinhoff, M. Hoppema, J. Olafsson, T. S. Arnarson, B. Tilbrook, T. Johannessen, A. Olsen, R. Bellerby, C. S. Wong, B. Delille, N. R. Bates, H. J. W. de Baar, Climatological mean and decadal change in surface ocean pCO_2, and net sea–air CO_2 flux over the global oceans. *Deep-Sea Res. II Top. Stud. Oceanogr.* **56**, 554–577 (2009).

34. O. Boucher, J. Servonnat, A. L. Albright, O. Aumont, Y. Balkanski, V. Bastrikov, S. Bekki, R. Bonnet, S. Bony, L. Bopp, P. Braconnot, P. Brockmann, P. Cadule, A. Caubel, F. Cheruy, F.

Codron, A. Cozic, D. Cugnet, F. D'Andrea, P. Davini, C. de Lavergne, S. Denvil, J. Deshayes, M. Devilliers, A. Ducharne, J. L. Dufresne, E. Dupont, C. Éthé, L. Fairhead, L. Falletti, S. Flavoni, M. A. Foujols, S. Gardoll, G. Gastineau, J. Ghattas, J. Y. Grandpeix, B. Guenet, L. E. Guez, E. Guilyardi, M. Guimberteau, D. Hauglustaine, F. Hourdin, A. Idelkadi, S. Joussaume, M. Kageyama, M. Khodri, G. Krinner, N. Lebas, G. Levavasseur, C. Lévy, L. Li, F. Lott, T. Lurton, S. Luyssaert, G. Madec, J. B. Madeleine, F. Maignan, M. Marchand, O. Marti, L. Mellul, Y. Meurdesoif, J. Mignot, I. Musat, C. Ottlé, P. Peylin, Y. Planton, J. Polcher, C. Rio, N. Rochetin, C. Rousset, P. Sepulchre, A. Sima, D. Swingedouw, R. Thiéblemont, A. K. Traore, M. Vancoppenolle, J. Vial, J. Vialard, N. Viovy, N. Vuichard, Presentation and evaluation of the IPSL-CM6A-LR Climate Model. *J. Adv. Model. Earth Syst.* **12**, e2019MS002010 (2020).

35. C. J. Smith, G. R. Harris, M. D. Palmer, N. Bellouin, W. Collins, G. Myhre, M. Schulz, J. C. Golaz, M. Ringer, T. Storelvmo, P. M. Forster, Energy budget constraints on the time history of aerosol forcing and climate sensitivity. *J. Geophys. Res. Atmos.* **126**, e2020JD033622 (2021).

36. M. Crippa, E. Solazzo, G. Huang, D. Guizzardi, E. Koffi, M. Muntean, C. Schieberle, R. Friedrich, G. Janssens-Maenhout, High resolution temporal profiles in the Emissions Database for Global Atmospheric Research. *Sci. Data* **7**, 121–137 (2020).

37. S. M. Jones, M. Hoggett, S. E. Greene, T. Dunkley Jones, Large Igneous Province thermogenic greenhouse gas flux could have initiated Paleocene-Eocene Thermal Maximum climate change. *Nat. Commun.* **10**, 5547 (2019).

38. K. Iyer, H. Svensen, D. W. Schmid, SILLi 1.0: A 1-D numerical tool quantifying the thermal effects of sill intrusions. *Geosci. Model Dev.* **11**, 43–60 (2018).

39. J. Lambert Jr., G. Simkovich, P. Walker Jr., The kinetics and mechanism of the pyrite-to-pyrrhotite transformation. *Metall. Mater. Trans. B* **29**, 385–396 (1998).

40. B. Fegley Jr., K. Lodders, A. Treiman, G. Klingelhöfer, The rate of pyrite decomposition on the surface of Venus. *Icarus* **115**, 159–180 (1995).

41. S. Chang, R. A. Berner, Coal weathering and the geochemical carbon cycle. *Geochim. Cosmochim. Acta* **63**, 3301–3310 (1999).

42. J. J. Sweeney, A. K. Burnham, Evaluation of a simple model of vitrinite reflectance based on chemical kinetics. *AAPG Bull.* **74**, 1559–1570 (1990).

43. J. P. Landwehrs, G. Feulner, M. Hofmann, S. Petri, Climatic fluctuations modeled for carbon and sulfur emissions from end-Triassic volcanism. *Earth Planet. Sci. Lett.* **537**, 116174 (2020).

44. M. Steinthorsdottir, C. Elliott-Kingston, K. L. Bacon, Cuticle surfaces of fossil plants as a potential proxy for volcanic SO_2 emissions: Observations from the Triassic–Jurassic transition of East Greenland. *Paleobiodivers. Paleoenviron.* **98**, 49–69 (2018).

45. P. Olsen, J. Sha, Y. Fang, C. Chang, J. H. Whiteside, S. Kinney, H.-D. Sues, D. Kent, M. Schaller, V. Vajda, Arctic ice and the ecological rise of the dinosaurs. *Sci. Adv.* **8**, eabo6342 (2022).

46. J. H. F. L. Davies, A. Marzoli, H. Bertrand, N. Youbi, M. Ernesto, U. Schaltegger, End-Triassic mass extinction started by intrusive CAMP activity. *Nat. Commun.* **8**, 15596 (2017).

47. S. M. Newby, J. D. Owens, S. D. Schoepfer, T. J. Algeo, Transient ocean oxygenation at end-Permian mass extinction onset shown by thallium isotopes. *Nat. Geosci.* **14**, 678–683 (2021).

48. C. Capitani, K. Petrakakis, The computation of equilibrium assemblage diagrams with Theriak/Domino software. *Am. Mineral.* **95**, 1006–1016 (2010).

49. T. J. B. Holland, R. Powell, An improved and extended internally consistent thermodynamic dataset for phases of petrological interest, involving a new equation of state for solids. *J. Metamorph. Geol.* **29**, 333–383 (2011).

50. Z. Doner, M. Kumral, I. H. Demirel, Q. Hu, Geochemical characteristics of the Silurian shales from the central Taurides, southern Turkey: Organic matter accumulation, preservation and depositional environment modeling. *Mar. Pet. Geol.* **102**, 155–175 (2019).

51. G. Hu, K. Dam-Johansen, S. Wedel, J. P. Hansen, Decomposition and oxidation of pyrite. *Prog. Energy Combust. Sci.* **32**, 295–314 (2006).

52. G. L. Foster, D. L. Royer, D. J. Lunt, Future climate forcing potentially without precedent in the last 420 million years. *Nat. Commun.* **8**, 14845 (2017).

53. G. Myhre, E. J. Highwood, K. P. Shine, F. Stordal, New estimates of radiative forcing due to well mixed greenhouse gases. *Geophys. Res. Lett.* **25**, 2715–2718 (1998).

54. S. Twomey, The influence of pollution on the shortwave albedo of clouds. *J. Atmos. Sci.* **34**, 1149–1152 (1977).

55. B. A. Albrecht, Aerosols, cloud microphysics, and fractional cloudiness. *Science* **245**, 1227–1230 (1989).

56. A. S. Ackerman, M. P. Kirkpatrick, D. E. Stevens, O. B. Toon, The impact of humidity above stratiform clouds on indirect aerosol climate forcing. *Nature* **432**, 1014–1017 (2004).

57. I. M. Held, M. Winton, K. Takahashi, T. Delworth, F. Zeng, G. K. Vallis, Probing the fast and slow components of global warming by returning abruptly to preindustrial forcing. *J. Clim.* **23**, 2418–2427 (2010).

58. Q. Jiang, F. Jourdan, H. K. Olierook, R. E. Merle, An appraisal of the ages of Phanerozoic large igneous provinces. *Earth Sci. Rev.* **237**, 104314 (2023).

59. L. Rosa, M. C. Rulli, K. F. Davis, P. D'Odorico, The water-energy nexus of hydraulic fracturing: a global hydrologic analysis for shale oil and gas extraction. *Earth's Future* **6**, 745–756 (2018).

60. M. S. Diamond, E. M. Stewart, michael-s-diamond/MetamorphicSulfur, version 20260513 [Software], Zenodo (2026); https://zenodo.org/records/20169473.

61. K. Evans, R. Powell, T. Holland, Internally consistent data for sulphur-bearing phases and application to the construction of pseudosections for mafic greenschist facies rocks in Na_2O–CaO–K_2O–FeO–MgO–Al_2O_3–SiO_2–CO_2–O–S–H_2O. *J. Metam. Geol.* **28**, 667–687 (2010).

62. T. Holland, R. Powell, An internally consistent thermodynamic data set for phases of petrological interest. *J. Metam. Geol.* **16**, 309–343 (1998).

63. T. J. B. Holland, E. C. R. Green, R. Powell, A thermodynamic model for feldspars in $KAlSi_3O_8$−$NaAlSi_3O_8$−$CaAl_2Si_2O_8$ for mineral equilibrium calculations. *J. Metam. Geol.* **40**, 587–600 (2022).

64. R. W. White, R. Powell, T. Johnson, The effect of Mn on mineral stability in metapelites revisited: New a–x relations for manganese-bearing minerals. *J. Metam. Geol.* **32**, 809–828 (2014).

65. E. Green, R. White, J. Diener, R. Powell, T. Holland, R. Palin, Activity–composition relations for the calculation of partial melting equilibria in metabasic rocks. *J. Metam. Geol.* **34**, 845–869 (2016).

66. T. Holland, R. Powell, Activity–composition relations for phases in petrological calculations: An asymmetric multicomponent formulation. *Contrib. Mineral. Petrol.* **145**, 492–501 (2003).
